# New biomarkers: prospect for diagnosis and monitoring of thyroid disease

**DOI:** 10.3389/fendo.2023.1218320

**Published:** 2023-07-21

**Authors:** Mirjana T. Macvanin, Zoran M. Gluvic, Bozidarka L. Zaric, Magbubah Essack, Xin Gao, Esma R. Isenovic

**Affiliations:** ^1^ Department of Radiobiology and Molecular Genetics, VINČA Institute of Nuclear Sciences - National Institute of the Republic of Serbia, University of Belgrade, Belgrade, Serbia; ^2^ Clinic for Internal Medicine, Department of Endocrinology and Diabetes, Zemun Clinical Hospital, Faculty of Medicine, University of Belgrade, Belgrade, Serbia; ^3^ Computer Science Program, Computer, Electrical and Mathematical Sciences and Engineering Division (CEMSE), King Abdullah University of Science and Technology (KAUST), Thuwal, Saudi Arabia; ^4^ Computational Bioscience Research Center (CBRC), King Abdullah University of Science and Technology (KAUST), Thuwal, Saudi Arabia

**Keywords:** thyroid disorders, thyroid cancer, biomarkers, mRNA, non-coding RNAs, miRNA, lncRNA, circRNA

## Abstract

After the metabolic syndrome and its components, thyroid disorders represent the most common endocrine disorders, with increasing prevalence in the last two decades. Thyroid dysfunctions are distinguished by hyperthyroidism, hypothyroidism, or inflammation (thyroiditis) of the thyroid gland, in addition to the presence of thyroid nodules that can be benign or malignant. Thyroid cancer is typically detected *via* an ultrasound (US)-guided fine-needle aspiration biopsy (FNAB) and cytological examination of the specimen. This approach has significant limitations due to the small sample size and inability to characterize follicular lesions adequately. Due to the rapid advancement of high-throughput molecular biology techniques, it is now possible to identify new biomarkers for thyroid neoplasms that can supplement traditional imaging modalities in postoperative surveillance and aid in the preoperative cytology examination of indeterminate or follicular lesions. Here, we review current knowledge regarding biomarkers that have been reliable in detecting thyroid neoplasms, making them valuable tools for assessing the efficacy of surgical procedures or adjunctive treatment after surgery. We are particularly interested in providing an up-to-date and systematic review of emerging biomarkers, such as mRNA and non-coding RNAs, that can potentially detect thyroid neoplasms in clinical settings. We discuss evidence for miRNA, lncRNA and circRNA dysregulation in several thyroid neoplasms and assess their potential for use as diagnostic and prognostic biomarkers.

## 1 Introduction

**Figure 1:**
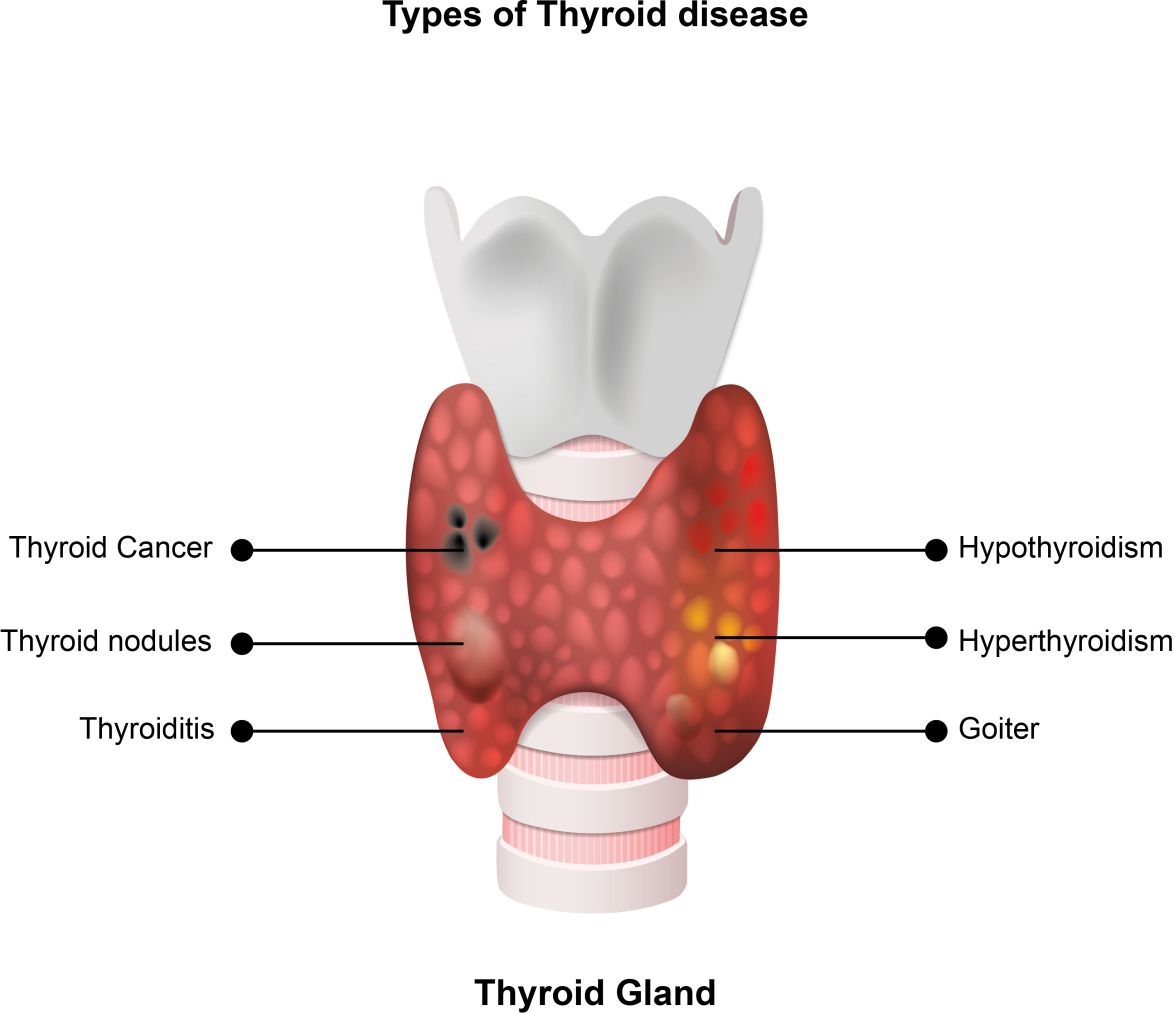
Types of thyroid diseases. Hypothyroidism is a medical condition in which the thyroid gland produces insufficient levels of thyroid hormones, and hyperthyroidism is a medical condition caused by high levels of thyroid hormones in the blood. Thyroid nodules are small lesions within the thyroid gland, and goiter is a thyroid gland enlargement that can be diffuse or nodular. Thyroid cancer is the most common endocrine malignancy and the ninth most common cancer. Depending on the origin, it is classified as differentiated thyroid cancer, medullary thyroid cancer, or anaplastic thyroid cancer. Some adaptations and new terminology exist in the 2022 WHO classification of thyroid tumors. Thyroiditis is inflammation of the thyroid gland, associated with normal, high or low levels of thyroid hormones in the blood.

Thyroid cancer is the most common endocrine malignancy and the ninth most common cancer overall (1). It is classified as differentiated thyroid cancer (papillary, follicular, or Hürthle cell), medullary thyroid cancer (MTC), or anaplastic thyroid cancer, depending on its origin (2–4). Differentiated thyroid cancer (DTC) originates from follicular thyroid cells and accounts for roughly 90% of thyroid cancers (2). When adequately treated, DTC has a favourable prognosis compared to other malignancies, with a 20-year survival rate approaching 90% (5). Types of thyroid diseases are presented on **Figure 1**. According to the 2022 World Health Organization (WHO) guidelines, the classification of thyroid neoplasms is based on histopathology and molecular pathogenesis. Some adaptations and new terminology, especially for malignant thyroid neoplasms, are implemented to improve their management (6).

**Figure 2:**
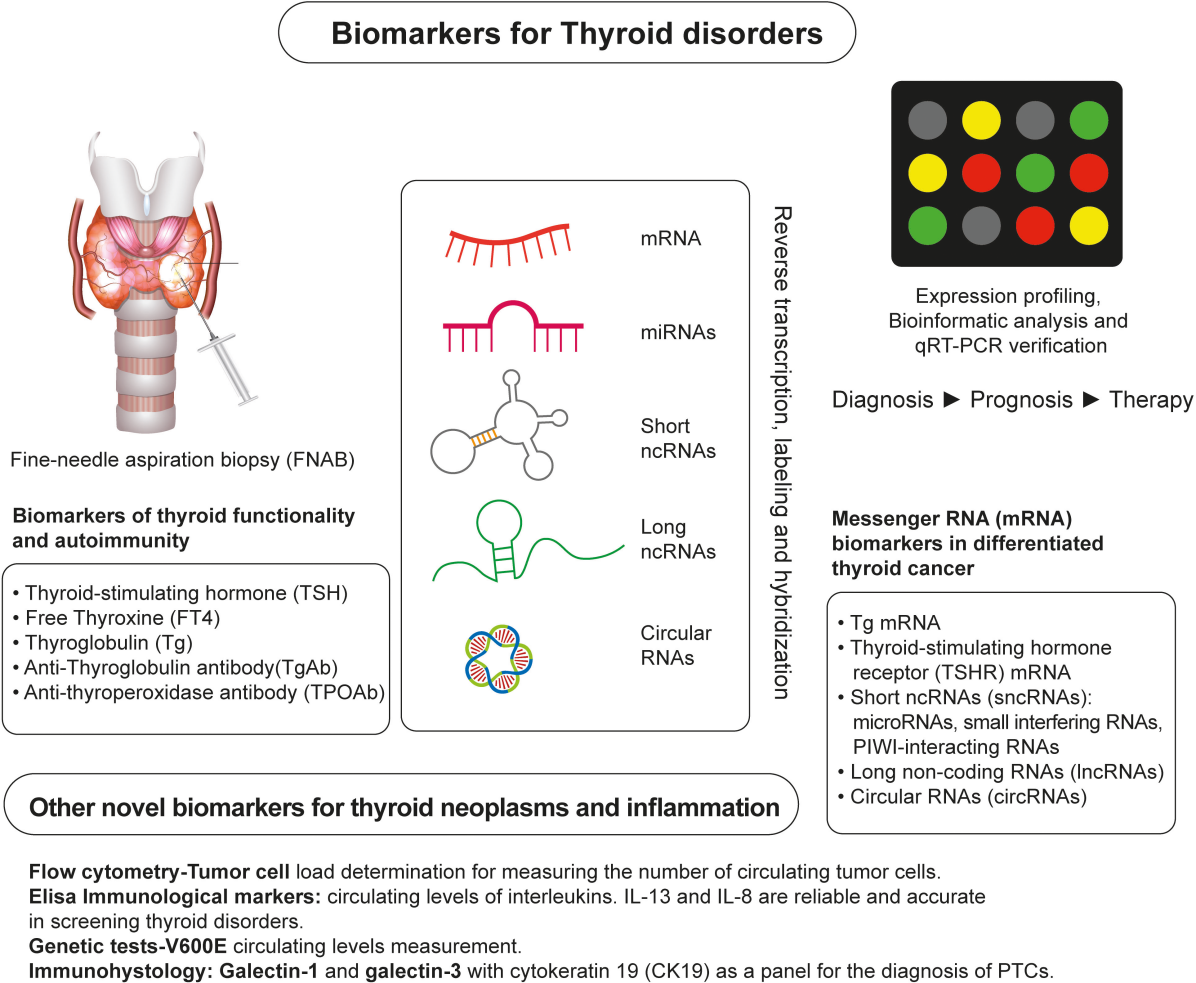
Overview of Identification of Biomarkers of Thyroid Neoplasms. A conventional approach for detecting and monitoring thyroid neoplasms relies on fine-needle aspiration biopsy (FNAB) and measurements of traditional biochemical markers, such as TSH, FT4, Tg, TgAb, and TPOAB. Novel RNA-based markers of thyroid neoplasms are identified by expression profiling of the thyroid gland tissue and/or blood samples of patients using high-throughput platforms for RNA analysis and identification, such as next-generation sequencing. Other emerging biomarkers of thyroid neoplasms are identified using flow cytometry, ELISA assays, genetic tests, and immunohistological analyses.

Nearly 50 years ago, biomarkers were first utilized to detect and manage malignant thyroid neoplasia (7, 8). Since then, several biomarkers have been confirmed as highly reliable in detecting malignant thyroid disorders and represent valuable tools used in the postoperative assessment of the effectiveness of surgical and radio-ablative procedures or chemotherapeutic treatment (9) (**Figure 2**). For thyroid cancer diagnosis, ultrasound-guided fine-needle aspiration biopsy (FNAB) and the cytological examination of the specimen are combined (10). However, this approach has limitations due to the small sample size (11) and the inability to characterize follicular lesions adequately. Sometimes it could be challenging to differentiate follicular variant of papillary thyroid carcinoma (FVPTC) or follicular thyroid carcinoma FTC) from benign thyroid neoplasms with a follicular growth pattern. Nondiagnostic, indeterminate, or suspicious lesions account for 15%-25% of cases investigated by US-guided FNAB, with 30% eventually revealing to be malignant (12, 13).

Furthermore, circulating biomarkers like calcitonin (CT), Tg, and TgAb are routinely used in postoperative monitoring. They cannot distinguish between benign and malignant neoplasms or low- and high-risk malignant lesions in the preoperative stage (10). Therefore, there is an urgent need to identify a molecular biomarker that can complement traditional imaging modalities in postoperative surveillance and assist cytology examination in indeterminate or follicular lesions preoperatively. This article reviews the present state of thyroid neoplasm biomarkers and their benefits and limitations. We are particularly interested in providing a complete update on recently discovered biomarkers with promising therapeutic uses for diagnosing and managing thyroid neoplasms.

### 1.1 Search strategy

We performed a systematic literature search on PubMed and MEDLINE for English and non-English articles with English abstracts published between 1990 and 2023. The top search terms were: thyroid disorders, thyroid neoplasms, thyroid cancer, biomarkers, mRNA, non-coding RNAs, miRNA, lncRNA, circRNA, and thyroid therapy. The search retrieved original peer-reviewed articles, further analyzed, focusing on emerging RNA and immunological biomarkers that could detect and manage thyroid neoplasms in the clinical setting. To avoid the exclusion of relevant studies, we adopted the “related articles” function of PubMed to identify other potentially relevant literature. We specifically focused on including the most recent findings published in the past five years.

## 2 Detection of messenger RNA biomarkers in circulating cells

The periodic determination of serum Tg levels in DTC patients is used to detect recurrent or metastatic disease (14). The method’s low sensitivity and the interference of TgAbs with the assay limit the use of Tg as a tumor marker (15). An alternative approach is monitoring mRNA levels for Tg (TG mRNA) and TSH receptor (TSH-R mRNA) in circulating thyrocytes. Circulating cells have been thought to be an indication of malignant potential. The detection and quantification of mRNAs only found in cancerous cells is an indirect method of determining the presence of cancerous cells in the bloodstream. This approach has promising clinical potential, according to research conducted over the last two decades (16).

### 2.1 Thyroglobulin mRNA as a potential biomarker for thyroid cancer

Tg mRNA detection in blood samples is proposed as a potential tumor biomarker for the following reasons: i) Malignant tumors shed cells that can be isolated from the peripheral circulation (13); ii) Tg mRNA is present in blood samples from DTC patients with known metastases (17). However, the assumption that Tg mRNA originates only from circulating thyroid cancer cells or micrometastases is questioned by experimental evidence showing that Tg mRNA is also present in benign thyroid neoplasms and healthy individuals (18, 19). Specifically, Ringel and colleagues (20) demonstrated that RT-PCR amplified Tg mRNA from blood samples of 77 patients who had undergone thyroidectomy for DTC, 68 of whom received levothyroxine (L-T4) for TSH suppression. For comparison, they show Tg mRNA detected with either thyroid bed or metastatic iodine-avid tissue in 26 of 33 patients (79%) on L-T4 therapy, and serum Tg detected in only 12 of these 33 patients (36%), which strongly argues in favor of increased sensitivity of Tg mRNA detection. However, Tg mRNA was detected by RT-PCR in healthy controls, suggesting that other sources of Tg mRNA may influence the specificity of detection. These findings indicate that small numbers of thyroid cells are present in the circulation and are most likely the source of Tg mRNA detectable using a highly sensitive method like RT-PCR (18). Other potential sources of Tg mRNA that can interfere with the specificity of detection have been suggested, such as lymphocytes (21) and renal cells (22). Also, increasing the number of amplification cycles can influence RT-PCR specificity, resulting in a significant increase in false-positive results in healthy subjects (23).

Several studies have found significant overlap in Tg mRNA values among various thyroid disorders and, in some cases, healthy controls (24–26). Illegitimate Tg transcription and splice variants that appear more common than previously thought were proposed to compromise the specificity of Tg mRNA detection (23, 27). Careful selection of primers to amplify Tg mRNA that avoids contamination with known splice variants may overcome this problem and significantly increase the assay’s sensitivity and specificity (28, 29). Other studies have pointed to additional problems (sample collection methodology, sample storage, RNA isolation, primer selection, and amplification cycles) discerning Tg mRNA originating from circulating malignant thyrocytes from other sources (23). Tg mRNA detection, for example, can occur in thyroid disorders such as thyroiditis, wherein thyroid tissue is destroyed. Inadequate evidence supports using Tg mRNA as a reliable preoperative screening tool. Existing evidence suggests that Tg mRNA detection may play an essential role in thyroid cancer surveillance in patients where serum Tg determination is of limited value due to the presence of TgAb prior to surgery.

### 2.2 The potential of TSHR mRNA as a thyroid cancer biomarker

Thyroid-stimulating hormone receptor (TSHR), mRNA has been studied as a thyroid cancer marker in pre-surgery diagnosis (30), detecting malignancy in indeterminate FNA results (31), monitoring thyroid cancer recurrence (28, 30, 31), and predicting tumor aggressiveness (32). Preoperative TSHR mRNA measurements yielded results comparable to FNAB specimen cytology (30, 33, 34). TSHR mRNA had a sensitivity of 76% and specificity of 96% in detecting malignancy in preoperatively diagnosed follicular lesions (33). The most important finding of this study is a transitory increase in TSHR mRNA levels in patients with goitres whose blood was drawn following FNAB (33). This result is especially significant because only a few studies investigating TSHR mRNA detection report when blood samples are taken, that is, before or after FNAB. For samples collected after FNAB of lesions suspicious of malignancy, artificially elevated levels of TSHR mRNA may be observed in patients with a high likelihood of malignancy (35).

A study analyzing the usefulness of TSHR-mRNA combined with neck ultrasonography (US) in the management of thyroid nodules with Bethesda III-V cytology reported sensitivity ranging from 33%-79%, depending on the classification of the lesions (Bethesda III-V) (31). Negative TSHR mRNA results combined with US results can reliably rule out cancer. Furthermore, after total thyroidectomy, TSHR mRNA levels are significantly reduced (30). Consistently elevated postoperative levels predict residual disease or disease recurrence. In patients with persistent disease and increased TgAb levels, the TSHR mRNA assay showed reasonable sensitivity (36).

The limitations of this method are the same as those described in the case of Tg mRNA. Numerous factors influence the sensitivity and reproducibility of detection, ranging from sample collection to primer selection. Another issue with using TSHR mRNA to detect thyroid cancer is that its presence in the bloodstream does not entirely reflect its expression in thyroid tissue, as the thymus, pituitary gland, kidney, heart, and retro-orbital tissues also reportedly express TSHR mRNA (37–39). For instance, in Graves’ ophthalmopathy, increased expression of TSHR in orbital fibroblasts and adipocytes is a critical step in the disease pathogenesis (38).

## 3 Non-coding RNAs as thyroid disease biomarkers

**Figure 3:**
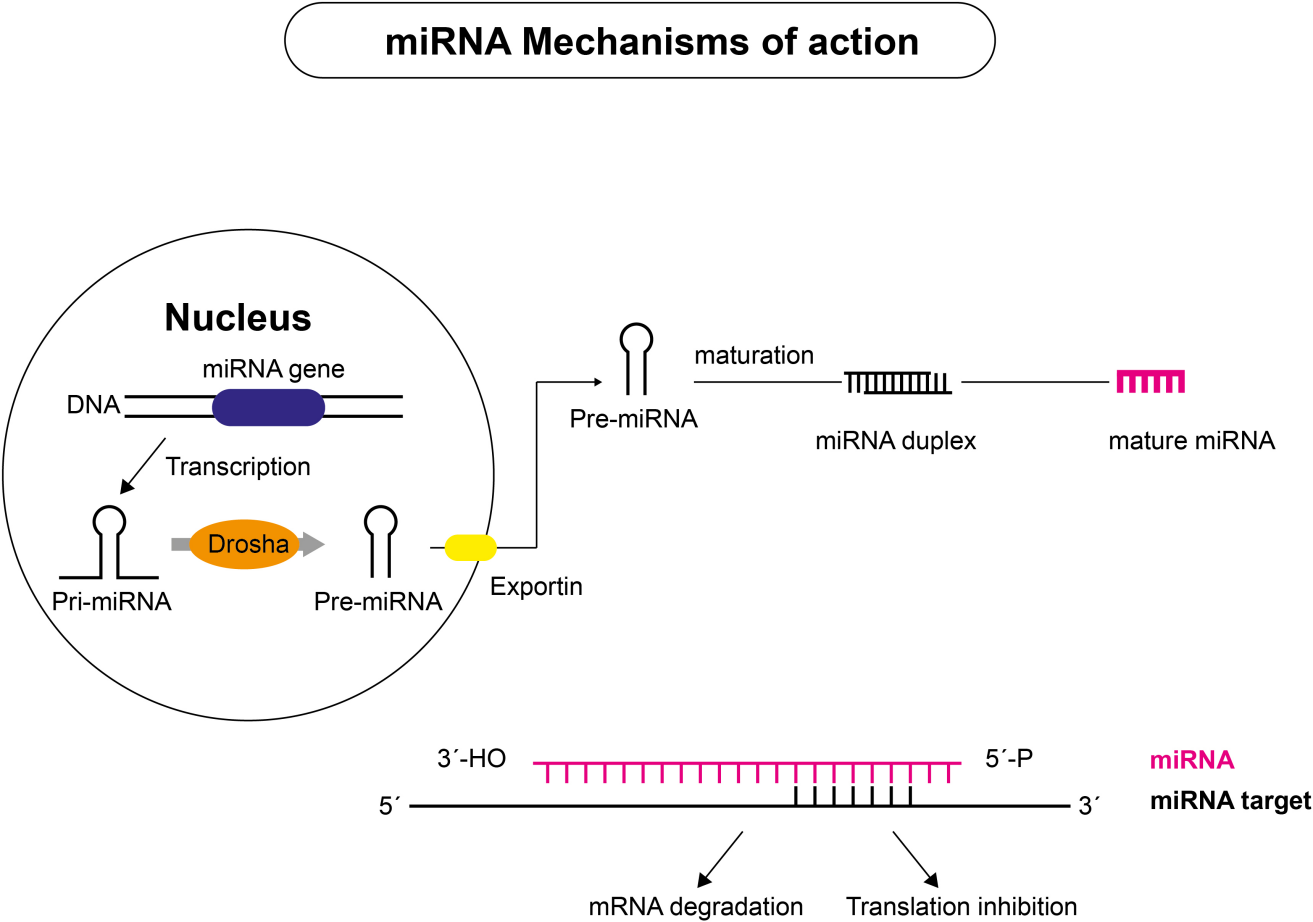
Mechanism of action of miRNAs. miRNAs are transcribed from DNA sequences into primary miRNAs (pri-miRNAs), which undergo endolytic processes to produce mature miRNAs. Pri-miRNAs are transcribed in the nucleus by RNA polymerase II and cut to approximately 70 nucleotide-long pre-miRNA molecules that are exported to the cytoplasm by the endoribonuclease DROSHA or by components of the splicing machinery. Mature miRNA duplexes are produced after further processing by the type III endoribonuclease DICER, which is associated with RNA-binding proteins. The mature miRNA guide strand joins with proteins to form the silencing complex, which binds to complementary sequences on target mRNA. Depending on the level of complementarity between miRNA and target mRNAs, two outcomes are possible: target mRNA slicing or translational inhibition, with subsequent target mRNA decay.

**Figure 4:**
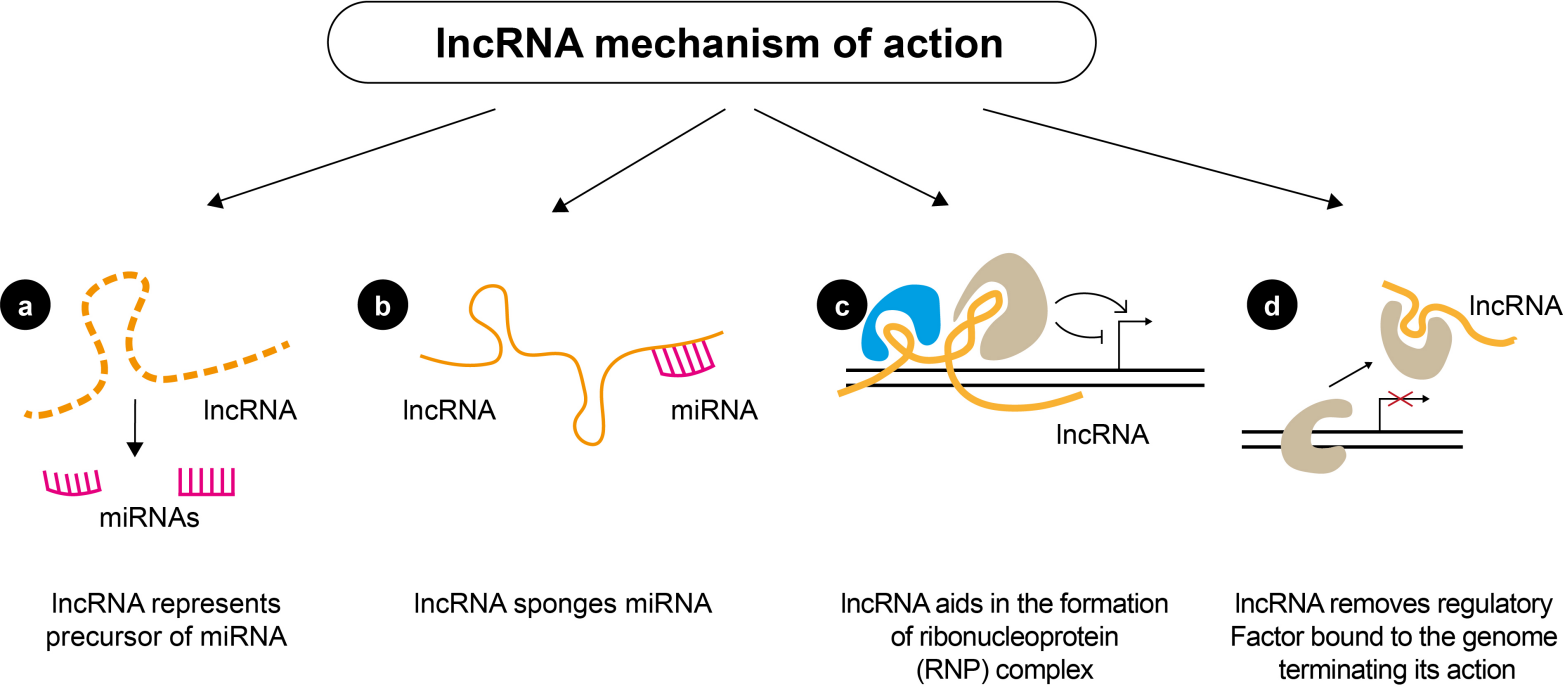
Mechanisms of action of lncRNAs. lncRNAs can regulate gene expression by **(A)** serving as precursors of miRNAs to affect the regulation of miRNAs directly; **(B)** acting as a sponge of miRNAs and inhibiting the degradation of mRNAs targeted by miRNAs; **(C)** lncRNAs can also function as scaffolds to form ribonucleoprotein (RNP) complexes; **(D)** lncRNA can inhibit the binding of a transcriptional regulatory factor, by directly interacting with them and acting as a “decoy”, which abolishes their action.

An increasing body of evidence demonstrates the crucial role of regulatory non-coding RNAs (ncRNAs) in initiating and progressing various diseases (20, 40–42). Non-coding RNAs account for nearly 98% of the human transcriptome and include a wide range of transcripts with diverse functions. Short ncRNAs (sncRNAs) and long ncRNAs (lncRNAs) are the two types of ncRNAs (43–45). Short ncRNAs have less than 200 nucleotides, including microRNAs (miRNAs), small interfering RNA, and PIWI-interacting RNAs (46). The short miRNA molecules (20-25 nucleotides) are involved in the post-transcriptional regulation of gene expression (**Figure 3**). They mainly interact with the 3′-untranslated region of the target mRNA, suppressing their translation through RNA-interference mechanisms. lncRNAs are ncRNAs longer than 200 nucleotides and represent a group of diverse molecules that regulate gene expression and cellular function and play a key role in tumorigenesis and tumor progression (47–49). lncRNAs serve as a precursor of miRNAs molecules and they can either directly or indirectly affect miRNAs via competitive binding, thus influencing gene expression at both the nuclear and cytoplasmic levels (**Figure 4**). circRNAs are covalently closed, circular RNAs formed through alternative back-splicing of protein-coding exons. Many ncRNAs were expressed differently in thyroid carcinoma tissues (50–53). Here we briefly summarize recent findings on ncRNAs relevant to the diagnosis and prognosis of thyroid neoplasms.

### 3.1 Specific microRNA (miRNA) expression profiles have been linked to malignant thyroid neoplasms

miRNAs are small, non-coding RNA molecules that regulate mRNA at the post-transcriptional level in various biological processes such as proliferation, apoptosis, and cell differentiation (54). Regulation of miRNA expression is an essential factor in tumor development and progression. miRNA expression profiles differ between malignant and healthy tissue (55), individual tumor subtypes, and primary and metastatic tumors. Tetzlaff and colleagues (56) found that specific miRNA expression profiles correlate with genetic mutations commonly found in DTCs. Unlike circulating mRNA, miRNAs are protected from nucleases in the bloodstream by binding to proteins or being encased in exosomes or microvesicles (57), and they can remain intact in paraffin-fixed tissue samples (58). Also, miRNAs are resistant to environmental conditions such as room temperature (59) and easily detectable in blood samples, suggesting they could work well as circulating biomarkers (60). In contrast to mRNA, miRNAs are thought to be markers of pathological processes rather than direct products of tumor cells.

Several studies aim to identify and analyze miRNA signatures distinguishing benign from malignant thyroid neoplasms which clinicians could use in postoperative monitoring (56, 61–67). Combining sequencing and quantitative RT-PCR revealed overexpressed miR-151-5p and miR-222 levels in PTC patients’ serum and tissue samples compared to goiter patients and healthy controls (61). The level of both miRNAs decreased after thyroidectomy to levels found in healthy individuals. Subsequent studies have identified other circulating miRNAs associated with PTC, such as miRNA-146b, miRNA-579, miRNA-95, miR-29b, miRNA-190, miR-25-3p and miR-451a (62, 63, 66) (**Table 1**). miR-146b is one of the most overexpressed miRNAs in PTC, and its expression positively correlates with the presence of a malignant thyroid neoplasm, making this miRNA a potential biomarker (71, 72). miR-146b-5p regulates cell proliferation and invasion and is up-regulated during the epithelial-mesenchymal transition, thus playing a role in PTC progression (96). Its importance as a possible biomarker is shown in multiple studies’ findings showing that several miRNA panels, including miR-146b, can accurately differentiate between malignant and benign lesions in FNAB specimens (97–100). Furthermore, circulating miR-146b levels have been indicated as a reliable and valuable serological marker for distinguishing between PTC and benign lesions (62, 101). Detection of miR-146b expression in various thyroid nodules by *in situ* hybridization analysis in formalin-fixed paraffin-embedded specimens (102) has demonstrated a remarkable diagnostic value in distinguishing PTC from poorly differentiated thyroid carcinoma, follicular adenomas, FTC, or anaplastic thyroid carcinoma (102).

**Table 1:** Potential non-coding RNA biomarkers of malignant thyroid neoplasms.

Name	Target	Function	References
miRNAs			
miR-222	p27 (CDKN1B)	Cell cycle regulation, proliferation, and migration	(68–70)
miR-146b	SMAD4, IRAK1, NFκβ, EGFR	Cell cycle, proliferation, migration, and invasion of PTC cells	(71, 72)
miR-25-3-p	SOCS4	Metastasis and invasion of tumor PTC/FTC cells	(66)
miR-221	p27 (CDKN1B)	Tumor suppressor; cell cycle regulator	(68–70, 168, 169)
miR-137	CXCL12	Thyroid cancer cell proliferation, migration, and invasion	(73, 74)
miR-486	KIAA1199 (CEMIP); Fibrillin-1	Invasion and metastatic potential of PTC cells	(75, 76)
miR-1179	HMGB1	Migration of PTC cells	(75, 77)
lncRNAs			
HOTAIR	miR-488-5p	PTC-associated lymph node metastasis	(78, 79)
TNRC6C-AS1	miR-513c-5p	Apoptosis of thyroid cancer cells	(80)
AB074169	KHSRP	Tumour cell proliferation	(81)
ZFAS1	miR-590-3p	Inhibition of apoptosis and stimulation of proliferation of PTC cells	(82)
AFAP1-AS1	miR-155-5p	ATC progression	(83)
TUG1	miR-145	Thyroid cancer cell proliferation	(84)
UNC5B-AS1	–	Proliferation, migration and invasion of PTC cells	(85)
LOC100129940-N	Wnt/*β*-catenin	Invasion and progression of PTC	(86)
LINC00313	miR-4429	Proliferation and migration of PTC cells	(87, 170)
HOXA-AS2	miR-520c-3p	PTC cell migration and invasion	(88)
MALAT1	miR-200-3p	Cell proliferation, apoptosis, migration, invasion, and autophagy formation in ATC	(89)
BANCR	TSHR	Development of malignant thyroid nodules	(90, 91)
circRNAs			
hsa-circ-u0058124	miR-218–5p	PTC proliferation and metastasis	(92)
circ-ITCH	miR-22-3p	Initiation and progression of PTC	(93)
circ-NEK6	miR-370-3p	Thyroid cancer progression	(94)
circ-BACH2	miR-139-5p	Cell proliferation, migration and invasion	(95)

PTC, Papillary thyroid cancer; ATC, Anaplastic thyroid cancer; CDKN1B, Cyclin Dependent Kinase Inhibitor 1B; SMAD4, SMAD Family Member 4, IRAK1, Interleukin 1 Receptor-Associated Kinase 1, NFκβ, Nuclear Factor Kappa-Light-Chain-Enhancer of Activated B Cells, EGFR, Epidermal Growth Factor Receptor; SOCS4, Suppressor Of Cytokine Signaling 4;CXCL12, C-X-C Motif Chemokine Ligand 12; CEMIP, Cell Migration Inducing Protein; HMGB1, High Mobility Group Box 1;HOTAIR, HOX Transcript Antisense RNA; KHSRP, KH-Type Splicing Regulatory Protein; ZFAS1, ZNFX1 Antisense RNA 1; AFAP1, AS1-Actin Filamentin-1 Antisense RNA; TUG1, Long non-coding RNA Taurine-Upregulated Gene 1; UNC5B, AS1- UNC5B Antisense RNA 1; LINC00313, Long Intergenic Non-Protein Coding RNA 313; HOXA, AS2- HOXA Cluster Antisense RNA 2; MALAT1, Metastasis Associated Lung Adenocarcinoma Transcript 1; BANCR, BRAF-Activated Non-protein Coding RNA; TSHR, Thyroid Stimulating Hormone Receptor; circ-ITCH, Circular RNA Itchy E3 Ubiquitin Protein Ligase; circ-NEK6, CircularRNA NEK6 (NIMA Related Kinase 6); circ-BACH2, Circular RNA BACH2 (BTB Domain And CNC Homolog 2).

Higher miR-146b-5p and miR-21 levels were associated with considerably lower PTC patient survival rates. MiR-146b-5p has been recommended as a diagnostic and prognostic marker for PTC due to its high expression in PTC but not in other tissues investigated (102). In addition, a study of thyroid cancer samples from The Cancer Genome Atlas (TCGA) revealed that miR-146b-5p-mediated regulation of the interleukin-1 receptor-associated kinase 1 gene (*IRAK1*) distinguishes the conventional PTC form (103). This finding is supported by a recent study reporting the involvement of NF-κB/IL6/STAT3 signaling cascade in controlling miR-146b-5p synthesis, whose increased levels downregulate the expression of pro-inflammatory mediators such as IRAK1 (104). It should also be noted that miR-146b deregulation was associated with aggressive tumor behavior in *BRAF*-positive clinical PTC specimens (105) and that patients with *BRAF* mutations exhibited increased miR-146b expression in comparison to *BRAF* wild-type controls (106).

miR-221 and miR-222, which share the same seed sequence, are also interesting, as they have altered expression in thyroid cancer based on several independent studies (68, 69) (see **Table 1**). The target of miR-221 and miR-222 in thyroid cancer is the mRNA of tumor suppressor and cell cycle regulator p27 (also known as CDKN1B) (70). Also, some downregulated miRNAs, directly or indirectly, regulate the proliferation and progression of thyroid cancer. For instance, a study of the miRNA expression profile in PTC showed that the expression of miR-137 was downregulated (73). miR-137 targets the mRNA of CXCL12, a chemokine up-regulated in PTC and associated with thyroid cancer cell proliferation, migration, and invasion (73, 74). The binding of CXCL12 to its receptor CXCR4 activates oncogenic pathways such as MAPK, ERK1, and ERK2 (107). These findings suggest that miR-137 functions as a tumor suppressor in PTC and support previous findings that the CXCL12/CXCR4 axis is a potential target for cancer treatment (73, 108) (**Table 1**).

miR-375 is significantly overexpressed in MTC compared to normal thyroid tissues, and there is a strong relationship between miR-375 tissue expression, tumor aggressiveness, and patient outcomes, implying a critical involvement in MTC pathogenesis (109, 110). Circulating miR-375 levels have been considered a promising prognostic marker for advanced MTC (111). A recent study reinforced the findings that serum miR-375 could be a diagnostic and prognostic marker of MTC, distinguishing between MTC patients and controls with a 92.6% sensitivity and a 97.6% specificity (112).

A 2022 study by Nieto and colleagues uses combinatorial mRNA and miRNA expression as prognostic indicators of thyroid cancer recurrence (75). They developed a risk score model based on detailed bioinformatics and experimental mRNA, miRNA, and somatic mutation analysis in recurrent tumors. They used total RNA sequencing data from 501 thyroid cancer samples, including 455 non-recurrent and 46 recurrent tumor specimens, retrieved from The Cancer Genome Atlas (TCGA). They conducted functional gene analyses in cell-based assays in multiple thyroid cell lines and assessed the prognostic value of the genes using the TCGA datasets (75). This study identified 40 mRNAs, 39 miRNAs, and 59 genetic variants as potential biomarkers of thyroid cancer recurrence. In particular, miR-486 and miR-1179 exhibited significant effects on inhibiting thyroid cancer cell migration *in vitro*, whereas deletion of miR-486 and miR-1179 increased *in vitro* cellular migration (75–77).

It is important to note that miRNA-based tests for classifying indeterminate thyroid nodules are already finding application in the clinical routine. The currently marketed panels combine tumor genotyping with gene and/or targeted miRNA expression profiling. To refine the risk stratification of cytologically indeterminate nodules, Interpace Diagnostics combines a genotyping panel (ThyGeNEXT) with a miRNA expression classifier optimized to have a high negative predictive value for thyroid cancer (113). For patients in Bethesda III or IV categories, tests can be performed on cells directly collected by FNAB or from smear slides prepared for cytologic evaluation (114). Samples are first evaluated by ThyGeNEXT, which represents a targeted DNA and RNA next-generation sequencing panel that includes gene fusions and hotspot mutations in several genes (115). A ThyraMIR panel, which uses quantitative RT-PCR to quantify the relative expression levels of 10 miRNAs, is used for further risk stratification. Thyroid nodules negative for ThyGeNEXT and ThyraMIR are considered low risk for non-invasive follicular thyroid neoplasm with papillary-like nuclear features (NIFTP)/cancer. MTC is recognized by the upregulation of specific miRNAs, such as miR-375, in the ThyraMIR panel (116). Concerning cancer probability, the results of both tests can be stratified into negative, moderate and positive, and may aid clinicians in deciding upon further clinical surveillance, lobectomy or thyroidectomy, respectively (113). Also, other miRNA gene expression classifiers have been developed to improve diagnostics of Bethesda III and IV categories, such as Rosetta GX Reveal (117) and the Brazilian mir-THYpe (118), but they require further extensive multicenter studies to confirm their performance (119).

### 3.2 Long non-coding RNAs as potential biomarkers for thyroid cancer

Long non-coding RNAs (lncRNAs) are non-coding RNAs with a length of more than 200 nucleotides that have been shown to mediate epigenetically controlled cancer progression mechanisms (120–122). lncRNAs regulate the expression of genes, including oncogenes or tumour-suppressive genes, either by binding directly to the target gene or by recruiting transcriptional regulators that promote chromatin modifications or DNA methylation (123, 124). lncRNAs can directly interact with proteins, such as transcription factors, DNA methyltransferases (DNMTs), RNA binding protein, and heterogeneous nuclear ribonucleoprotein (hnRNP) (120, 125, 126), to regulate their function in cancer progression (127). lncRNAs can also act by making complexes with miRNA, mRNA, or proteins to control the functions of the target protein in the cytoplasm (128). In addition, a recent discovery unveiled a new regulatory mechanism involving lncRNAs, in which lncRNAs function as a competitive endogenous RNA (ceRNA) by competing with other transcripts for shared miRNAs (129). In the ceRNA network, lncRNAs can act as miRNA sponges, increasing the expression of downstream mRNA.

Several studies have determined the expression of lncRNA alteration during PTC tumorigenesis (130). For instance, the lncRNA HOTAIR (HOx Transcript Antisense RNA), which regulates the miR-488-5p/NUP205 axis, was overexpressed in the serum of PTC patients with lymph node metastasis (78, 79). Therefore, determining lncRNA-HOTAIR in PTC patients’ tissue samples has been recommended as a biomarker for PTC prognosis (131) (**Table 1**). Microarray analysis identified several differentially expressed lncRNAs related to thyroid cancer, such as TNRC6C-AS1, that can cause DNA demethylation via the Hippo signaling pathway, promoting apoptosis of thyroid cancer cells (80, 132). AB074169, which has a role in cell proliferation, was significantly down-regulated in PTC, suggesting that this lncRNA may have an anticancer role in PTC (81). In another study, Tong and colleagues demonstrated that the increased expression of lncRNA ZFAS1 contributed to the progression of PTC by inhibiting apoptosis and stimulating the proliferation of PTC cells (82). Subsequent research has identified several other lncRNAs whose altered expression is involved in thyroid cancer cell metastasis and invasion, including AFAP1-AS1 (83), TUG1 (84), UNC5B-AS1 (85), LOC100129940-N (86), LINC00313 (87), and HOXA-AS2 (88). The lncRNAs MALAT1, HOTAIR, and BANCR are significant factors in the initiation and progression of thyroid cancer and are proposed as biomarkers for early detection and diagnosis (89–91, 133) (**Table 1**).

Even though the expression profile of lncRNAs in thyroid cancer has yet to be fully characterized and validated, more and more studies suggest lncRNAs as promising novel thyroid cancer biomarkers. However, further research and clinical validation are required to confirm its role in thyroid cancer progression.

### 3.3 Circular RNAs

**Figure 5:**
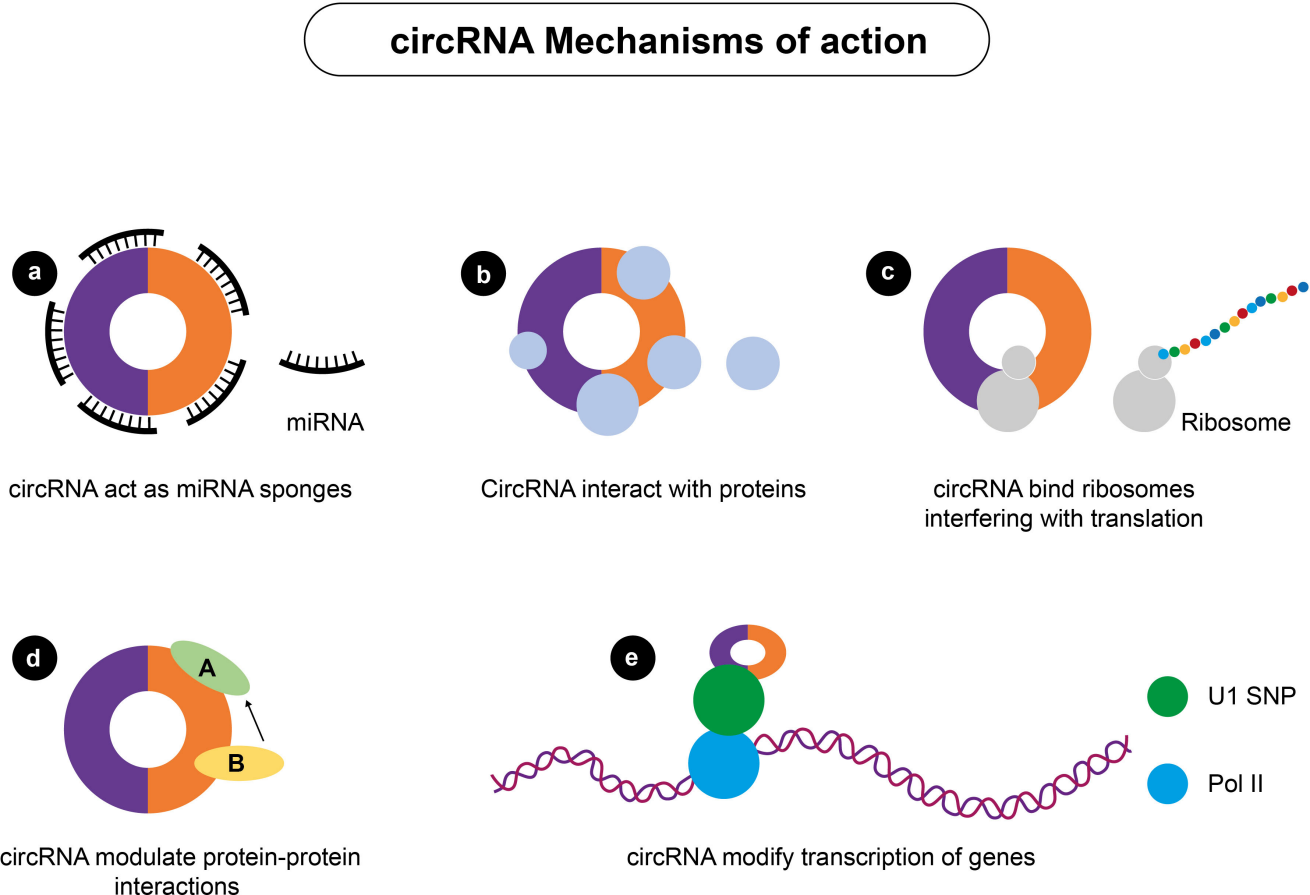
Mechanisms of actions of circRNA. circRNA can regulate gene expression by **(A)** acting as miRNA sponges; **(B)** interacting with various cellular proteins; **(C)** interfering with protein translation; **(D)** modulating protein-protein interactions; and **(E)** modifying the transcription of genes.

circRNAs with one or more miRNA-binding sites can act as RNA sponges, binding miRNAs and regulating the expression of miRNA-repressed downstream target genes (**Figure 5**) (134). Recently, several novel regulatory networks containing circRNAs that may serve as potential PTC biomarkers were discovered. For example, Yao and colleagues show that hsa-circ-u0058124 (Homo sapiens circRNA u0058124) promotes PTC cell proliferation, tumorigenicity, and metastasis and is associated with poor prognosis in PTC patients (92) (**Table 1**). has-circ-u0058124 regulates the expression of miR-218–5p target gene *NUMB* and inhibits the NOTCH3-GATAD2A signaling axis *in vitro* and *in vivo* (92). The hsa-circ-0058124/NOTCH3/GATAD2A axis is critical for PTC tumorigenesis and invasiveness. This study identifies a novel potential biomarker panel that may represent a target for therapeutic intervention in PTC progression (92). Another study by Wang and colleagues shows that the expression of circRNAs is lower in PTC tissues than in normal adjacent tissues and that the circ-ITCH/miR-22-3p/CBL/β-catenin pathway is involved in the initiation and progression of PTC (93). Chen and colleagues also show that circ-NEK6 targets miR-370-3p and promotes thyroid cancer progression by activating the Wnt signaling pathway (94) (**Table 1**). Cai and colleagues also show that repression of circ-BACH2 expression in PTC cells reduces cell proliferation, migration, and invasion, implying that circ-BACH2 is a novel oncogenic RNA that can serve as a PTC marker (95). In addition to these studies, a study published in 2021 used RNA deep sequencing to determine the expression patterns of circRNAs in PTC and identified several other circRNAs as promising and effective PTC biomarkers (135).

### 3.4 Clinical limitations of mRNAs and non-coding RNAs as thyroid neoplasm biomarkers

Although substantial research focused on examining circulating mRNA as potential diagnostic biomarkers of thyroid neoplasms, Tg and TSHR mRNA have not been widely accepted as accurate and valid biomarkers of thyroid neoplasms. There is insufficient evidence that Tg mRNA determination can be used as a preoperative screening tool, even though some reports suggest that the results may be comparable to serum Tg measurements, especially in TgAb-positive patients. Conflicting results in the literature may be due to methodological differences such as sample handling, mRNA isolation, primer design and number of amplification cycles. In addition, illegitimate Tg and TSH mRNA transcription may confound results. For instance, cell shedding resulting in Tg mRNA detection may occur in benign thyroid disorders such as thyroiditis, where destruction of thyroid tissue occurs. In the case of TSH mRNA, its detection may reflect extra-thyroidal mRNA expression since TSHR mRNA expression has been documented in multiple organs, such as the thymus, pituitary, retro-orbital tissues, heart and kidney.

Non-coding RNAs have gained momentum as a novel research target, with several promising miRNA, lncRNA and circRNA candidates identified and studied as potential diagnostic biomarkers of thyroid neoplasms. In particular, exciting progress has been achieved with the clinical application of commercially available miRNA-based panels, such as ThyraMIR (developed by Interpace Diagnostics). Also, evidence suggests several novel lncRNAs and circRNAs as potential diagnostic biomarkers of thyroid disease. However, further large-scale studies are necessary to validate promising experimental findings and examine the potential for their translation into the clinical setting.

## 4 Additional emerging biomarkers of thyroid neoplasms

Tumour cell load determination, immunological markers, circulating levels of BRAF V600E, and galectin were the most promising biomarkers for thyroid neoplasms diagnosis. Tumour load is determined using flow cytometry, which counts the number of circulating tumor cells in patients with metastatic medullary thyroid cancer (136). This method assumes that aggressive tumors shed more cells in the circulation, making circulating tumor cell enumeration a reasonable approach for assessing metastatic potential. In this regard, Xu and colleagues reported that higher tumor cell count signifies poorer outcomes in patients with metastatic medullary cancer (137). However, they found no statistically significant differences between patients with metastatic DTC and surgically treated controls with no evidence of disease recurrence (137). Angell and colleagues (138) also proposed measuring circulating levels of myeloid-derived suppressor cells to indicate DTC risk and extent because mean levels of these cells were higher in patients with DTC than those with benign TN and increased with the TN malignancy stage. The flow cytometry-based techniques are well-suited for postoperative monitoring after total thyroidectomy, where detecting a significant number of circulating cells could suggest the presence of residual disease or its recurrence. Studies in larger cohorts of patients are required to validate the correlation between a load of circulating tumor cells and their malignant potential and establish the quantitative cut-off values for the detection of circulating cells in the diagnosis and surveillance of DTC.

Thyroid neoplasms have been studied using circulating immunological biomarkers as potential biomarkers. Multiplex ELISA immunoassays have been used to assess interleukins as potential biomarkers that distinguish between individuals with thyroid neoplasms and healthy individuals (139). Compared to healthy controls, patients with benign and malignant TN had significantly higher levels of several interleukins (IL-6, IL-7, IL-10, and IL-13) and considerably lower levels of IL-8. A two-marker panel combining IL-13 and IL-8 showed high accuracy in screening for thyroid neoplasms (139). Also, multiplex ELISA immunoassay revealed an association between DTC recurrence and levels of soluble FAS ligand involved in the induction of apoptosis (140) and interferon α (141). This finding suggests a potential role of soluble FAS ligands in DTC surveillance. Recently, Lu and colleagues also used the flow cytometry approach to monitor the expression of T cell subsets and plasma cytokines in a cohort of 191 patients, including 79 patients with PTC and 58 with TNs. The study reported high activities of CD8^+^HLA-DR^+^and CD8^+^CD38^+^, together with increased levels of TNF-α in the PTC group compared to healthy controls. They proposed using a combination of immunological biomarkers such as CD8+HLA-DR+, CD8+CD38+, and TNF-α as valuable biomarkers for the early detection of PTC (142).

Another potential biomarker of thyroid neoplasms is *BRAF* V600. BRAF (the B-isoform of Raf kinase) oncogene is serine–threonine kinase and activator of the MAPK signaling cascade. Most mutations were discovered in exon 15, where a valine to glutamic acid substitution at residue 600 results (V600E) from a T-to-A transversion at nucleotide 1799. This mutation leads to constitutive activation of BRAF kinase and chronic stimulation of the MAPK pathway and is tumorigenic (143). Most PTC contain mutations in the BRAF gene, seen in 30% to 77% of these carcinomas in different ethnicities (144). The *BRAF* V600E mutation accounts for 99% (145), while other variants (like K601E) are present in 1%–2% of thyroid malignancies (146). *BRAF* somatic mutations have been reported in many human cancers, with the highest frequency in melanoma and thyroid cancer (147, 148).

According to histological subtype analysis, it was discovered that circulating *BRAF* V600E is more common in conventional PTC (72%) than the follicular variant (54%) (149). *BRAF V600E* somatic mutation evaluation in FNAB specimens is a powerful diagnostic tool for PTC. It was suggested that measuring *BRAF* V600E levels in the blood might be used instead of invasive procedures like biopsy or FNAB (150). *BRAF* V600E is one of the most represented somatic mutations in PTC; it is an appropriate marker for this disease (146).

The prognostic significance of the *BRAF* V600E mutation was investigated on 102 PTC patients who were diagnosed between 1985 and 1992. The authors analyzed the presence of the *BRAF* V600E mutation and correlated the presence of the mutation with the outcome and other clinicopathological features of PTC patients. The survival trend of PTC patients was analyzed according to the presence or absence of *BRAF* V600E mutation in tumor tissue. This 15-yr follow-up study showed that *BRAF* V600E-positive PTC patients have worse outcomes in terms of persistent disease and a lower survival rate (151). Nevertheless, multivariate analysis revealed that *BRAF* V600E mutation is a poor prognostic factor independent from other prognostic features (age, tumor size, vascular invasion).

In a large multicenter study, Tao et al. investigated the impact of lymph node metastasis (LNM) on PTC mortality with *BRAF* gene status in 2638 persons. Conventional PTC (CPTC) is the most common histological type, accounting for 70-75% of all PTC (152). The authors investigated the relationship between LNM and *BRAF* mutations in PTC patients. The probability of mortality in PTC patients with LNM has been shown to be closely associated with *BRAF* status. This is particularly evident in CPTC, where LNM had no effect on mortality in individuals with wild-type *BRAF* but significantly increased mortality in those with *BRAF* gene mutation (153). These findings contradict the long-held belief that LNM increases mortality in all PTC patients. Mortality is associated with the *BRAF* V600E mutation.

In a study by Damiani et al. (154), the prognostic potential of *BRAF* V600 was evaluated in a prospective study where 160 PTC patients were enrolled and were submitted to total thyroidectomy with a 2-10 years follow-up. According to the study’s findings, neither in patients who underwent radioiodine ablation treatment (RAI) nor in those who received more conventional care illness persistence did not correspond with the somatic *BRAF* V600E mutation. According to the findings of this study, BRAF V600E cannot be used as an independent predictive tool.

In 2012, the study was performed on 107 PTC patients in the Korean population. *BRAF* V600E mutation was present in 79% of the patients included in the study. The authors showed that in the population of Korean patients, *BRAF* V600 mutation does not correlate with prognostic factors such as extrathyroidal extensions, multifocality, tumor size, gender, age and lymph node metastases. This study concludes that in the Korean population of PTC patients, *BRAF* V600E does not indicate the aggressiveness of the tumor and that it has no prognostic value (155).

Because of inconsistent results in published articles, the relevance of *BRAF* V600E as a prognostic biomarker is controversial, and the analysis of *BRAF* mutation as a single, independent prediction factor cannot be employed in clinical practice (156).

Galectin family proteins, such as galectin-1 and galectin-3, have been considered for a long time as reliable histological markers of thyroid neoplasms since their mRNA expression levels are significantly altered in PTC (157). Proteomic profiling has also suggested galectin-1 as a potential thyroid cancer biomarker (158). In a recent study, Fanfone and colleagues aimed to develop imaging probes for non-invasive diagnosis of thyroid cancer. They identified the peptide (P7) that explicitly targeted galectin-1 by phage display and coupled it to imaging probes, such as near-infrared dye (CF770) or ultra-small superparamagnetic particles of iron oxide (USPIO), for non-invasive detection of galectin-1 expression in PTC by fluorescence or magnetic resonance imaging. The peptide-functionalized imaging probe showed an exceptional specificity (100%) and a high sensitivity (75%) of PTC detection, confirming the ability of this peptide to discriminate between malignant and benign TNs (159). Galectin-3, a carbohydrate-binding protein involved in tumor progression and metastasis (160), has also been investigated as a PTC tissue marker due to its specificity in the differential diagnosis of thyroid cancer (161, 162). Recently, the diagnostic utility of galectin-1 and galectin-3, alone or in combination with TPO, HBME-1 (Hector Battifora Mesothelial-1), and CK19 (cytokeratin 19), in benign and malignant TNs, was investigated to determine the utility of each marker or their combination for the accurate and reliable preoperative diagnosis of thyroid cancer (163). HBME1 is a monoclonal antibody that acts on the microvillous surface of mesothelial cells and is expressed explicitly in thyroid cancers but not benign lesions (164, 165). CK19 is an immunohistochemical stain expressed strongly in thyroid cancers (165, 166). According to the findings reported by Arcolia and colleagues, the combination of galectin-3 with CK19 and HBME1 is the most informative marker panel for the diagnosis of PTCs, with high specificity (97%) and sensitivity (95%) (163).

Finally, it should be noted that conventional diagnostic methods are not only limited to distinguishing between benign and malignant thyroid neoplasms but also distinguishes between different benign thyroid disorders. Hence, there is a need to identify novel biomarkers that discriminate between benign conditions with similar clinical presentations, such as destructive thyroiditis, which leads to thyrotoxicosis, and Graves’ disease. In a recent cross-sectional study, Fujita and colleagues used liquid chromatography-tandem mass spectrometry to measure serum diiodotyrosine (DIT) and monoiodotyrosine (MIT) levels to identify such biomarkers (167). They found serum DIT and MIT levels were significantly higher in destructive thyroiditis patients than in Graves’ disease patients, and serum DIT levels had high diagnostic accuracy (sensitivity, 84.6%; specificity, 77.3%; P = 0.001), implying that serum DIT levels may serve as a novel diagnostic biomarker for differentiating destructive thyroiditis from Graves’ disease (167).

## 5 Prospects and challenges for the clinical application of novel thyroid neoplasms biomarkers in the future

Next-generation sequencing (NGS) has become an irreplaceable, highly efficient, and accurate diagnostic tool that has revolutionized the diagnosis and management of thyroid cancer, allowing for the development of future personalized treatments. We are seeing exciting progress in discovering and validating novel biomarkers for thyroid neoplasms, with an ever-expanding list of biomarkers to establish the diagnosis, guide targeted therapy, predict clinical outcomes, and tumor response to therapy. Non-coding RNAs such as microRNAs, lncRNAs, and circRNAs appear to be the most promising markers. In addition, novel immunohistochemical (IHC) marker panels that are available, easy to use in the clinical setting, and cost-effective are also of particular interest. Immunotherapeutic approaches to thyroid cancer treatment are still being investigated, and future advances in this research field will complement the ongoing validation of novel biomarkers to ensure their appropriate application in clinical practice.

## 6 Conclusions

Due to the rapid advancement of high-throughput molecular biology techniques, it is now possible to identify novel biomarkers for thyroid neoplasms that can supplement or replace existing biomarkers. Current circulating biomarkers, such as Tg or TgAb, are routinely used in postoperative monitoring; however, at the preoperative stage, they cannot distinguish between benign and malignant neoplasms or between low- and high-risk malignant lesions. Several new targets are emerging as potentially useful prognostic circulating biomarkers for thyroid cancers. Evidence for miRNA, lncRNA and circRNA dysregulation in several thyroid neoplasms and their potential use as sensitive diagnostic and prognostic biomarkers is particularly interesting. However, the data currently available in the literature is derived from small-scale clinical studies in specific patient cohorts. Thus, further validation is required to demonstrate their potential for clinical application. Large-scale studies are needed to confirm and validate novel biomarkers in the diagnosis, prognosis, and surveillance of thyroid neoplasms, such as miRNAs, lncRNAs, and circRNAs.

## Author contributions

MM-conceived and wrote the article. BZ-wrote the article. ZG wrote the article, and EI, ME, and XG wrote and critically reviewed the article. All authors contributed to the article and approved the submitted version.
